# Completion of isoniazid preventive therapy among human immunodeficiency virus positive adults in urban Malawi

**DOI:** 10.5588/ijtld.17.0370

**Published:** 2018-03

**Authors:** D. Thindwa, P. MacPherson, A. T. Choko, M. Khundi, R. Sambakunsi, L. G. Ngwira, T. Kalua, E. L. Webb, E. L. Corbett

**Affiliations:** * Malawi-Liverpool-Wellcome Trust Clinical Research Programme, Blantyre, Malawi; † Department of Infectious Disease Epidemiology, Imperial College London, London; ‡ Department of Clinical Sciences, Liverpool School of Tropical Medicine, Liverpool; § Department of Public Health and Policy, University of Liverpool, Liverpool; ¶ Infectious Disease Epidemiology Department, London School of Hygiene & Tropical Medicine (LSHTM), London, UK; # Department of HIV/AIDS, Ministry of Health, Lilongwe, Malawi; ** Clinical Research Department, LSHTM, London, UK

**Keywords:** tuberculosis, loss to follow-up, risk factors, prospective, sub-Saharan Africa

## Abstract

**SETTING:**

Despite worldwide scale-up of human immunodeficiency virus (HIV) care services, relatively few countries have implemented isoniazid preventive therapy (IPT). Among other programmatic concerns, IPT completion tends to be low, especially when not fully integrated into HIV care clinics.

**OBJECTIVE:**

To estimate non-completion of 6-month IPT and its predictors among HIV-positive adults aged ⩾16 years.

**DESIGN:**

A prospective cohort study nested within a cluster-randomised trial of TB prevention was conducted between February 2012 and June 2014. IPT for 6 months was provided with pyridoxine at study clinics. Non-completion was defined as loss to follow-up (LTFU), death, active/presumptive TB or stopping IPT for any other reason. Random-effects logistic regression was used to determine predictors of non-completion.

**RESULTS:**

Of 1284 HIV-positive adults initiated on IPT, 885/1280 (69.1%) were female; the median CD4 count was 337 cells/μl (IQR 199–511); 320 (24.9%) did not complete IPT. After controlling for antiretroviral treatment status, IPT initiation year, age and sex, non-completion of IPT was associated with World Health Organization stage 3/4 (aOR 1.76, 95%CI 1.22–2.55), CD4 count 100–349 cells/μl (aOR 1.93, 95%CI 1.10–3.38) and any reported side effects (aOR 22.00, 95%CI 9.45–46.71).

**CONCLUSION:**

Completion of IPT was suboptimal. Interventions to further improve retention should target immunosuppressed HIV-positive adults and address side effects.

## Abstract (FR)

**CONTEXTE:**

En dépit de l'expansion mondiale des services de prise en charge de l'infection par le virus de l'immunodéficience humaine (VIH), assez peu de pays ont mis en œuvre le traitement préventif par isoniazide (IPT) en routine. Parmi d'autres préoccupations programmatiques, l'achèvement de l'IPT tend à être faible, surtout quand il n'est pas totalement intégré dans les centres de traitement du VIH.

**OBJECTIF:**

Estimer le niveau de non-achèvement des 6 mois d'IPT et ses facteurs de prédiction parmi des adultes âgés de ⩾16 ans positifs au VIH.

**SCHÉMA:**

Une étude prospective de cohorte au sein d'un essai randomisé en grappes de prévention de la tuberculose (TB) a été réalisée entre février 2012 et juin 2014. Un IPT de 6 mois a été fourni avec de la pyridoxine dans les centres de l'étude. Le non achèvement a été défini comme une perte de vue, un décès, une TB active/présumée ou un arrêt de l'IPT pour n'importe quelle autre raison. Une régression logistique à effets aléatoires a été utilisée pour déterminer les facteurs de prédiction de non achèvement.

**RÉSULTATS:**

Sur 1284 adultes positifs au VIH mis sous IPT, 885/1280 (69,1%) étaient des femmes et le CD4 médian a été de 337 cellules/μl (intervalle interquartile 199–511). Des 1284 adultes positifs au VIH, 320 (24,9%) n'ont pas achevé l'IPT. Après contrôle du statut du traitement antirétroviral, de l'année de mise en route de l'IPT, de l'âge et du sexe, le non achèvement de l'IPT a été associé au stade 3–4 de l'Organisation Mondiale de la Santé (ORa 1,76 ; IC95% 1,22–2,55), le nombre de CD4 100–349 cellules/μl (ORa 1,93 ; IC95% 1,10–3,38) et tout effet secondaire rapporté (ORa 22,00 ; IC95% 9,45–46,71).

**CONCLUSION:**

L'achèvement de l'IPT a été sousoptimal. Les interventions visant à améliorer la rétention devraient cibler les adultes immunodéprimés positifs au VIH et aborder le problème des effets secondaires.

## Abstract (SP)

**MARCO DE REFERENCIA:**

Pese a la ampliación mundial de escala de los servicios relacionados con la infección por el virus de la inmunodeficiencia humana (VIH), relativamente pocos países han introducido el tratamiento preventivo con isoniazida (IPT) en la práctica corriente. Entre otras dificultades programáticas, se observa una baja tasa de compleción del IPT, sobre todo cuando no se encuentra plenamente integrado en los consultorios de atención de la infección por el VIH.

**OBJETIVO:**

Calcular la tasa de tratamientos incompletos con un esquema de TPI de 6 meses y definir los factores asociados, en adultos a partir de los 16 años de edad, positivos frente al VIH.

**MÉTODO:**

Se llevó a cabo un estudio de cohortes prospectivo, anidado en un ensayo clínico aleatorizado por conglomerados sobre la prevención de la tuberculosis (TB), de febrero del 2012 a junio del 2014. El IPT con piridoxina se suministró durante 6 meses en los consultorios del estudio. La falta de compleción del tratamiento se definió como pérdida durante el seguimiento, muerte, TB activa confirmada o su presunción o interrupción del IPT por cualquier otra razón. Con un modelo de regresión logística de efectos aleatorios se determinaron los factores pronósticos de un tratamiento incompleto.

**RESULTADOS:**

Se inició el IPT en 1284 adultos positivos frente al VIH; 885 de 1280 eran de sexo femenino (69,1%) y la mediana de la cifra de linfocitos CD4 fue 337 células/μl (amplitud intercuartílica 199–511). No completaron el IPT 320 de los 1284 pacientes (24,9%). Tras ajustar con respecto a la situación frente al tratamiento antirretrovírico, el año de iniciación del IPT, la edad y el sexo, la falta de compleción del tratamiento se asoció con el estado 3 o 4 de la enfermedad según la clasificación de la Organización Mundial de la Salud (OR ajustado [ORa] 1,76; IC95% 1,22–2,55), la cifra de linfocitos CD4 de 100–349 células/μl (ORa 1,93; IC95% 1,10–3,38) y la notificación de algún efecto secundario (ORa 22,00; IC95% 9,45–46,71).

**CONCLUSIÓN:**

La tasa de compleción del IPT fue deficiente. Las intervenciones encaminadas a mejorar la retención en la atención se deben dirigir a los adultos positivos frente al VIH con inmunodepresión y deben abordar los efectos secundarios.

TUBERCULOSIS (TB) IS A GLOBAL public health threat, with a quarter of the population estimated to be latently infected worldwide.^1^ In 2015, the World Health Organization (WHO) estimated that there were 10.4 million incident TB cases and 1.4 million TB deaths globally.^2^ Approximately 11–13% of incident TB cases are co-infected with the human immunodeficiency virus (HIV).^2–4^ HIV-associated TB is highest in the WHO African Region, with HIV prevalence in TB patients exceeding 50% in some parts of southern Africa.^2^,^5^,^6^

Isoniazid (INH) preventive therapy (IPT) reduces the overall risk of TB in HIV-infected individuals by 35%, with greater reduction among tuberculin skin test (TST) positive individuals (pooled relative risk of 52%).^7^ When IPT is combined with antiretroviral treatment (ART), there is a multiplicative protective effect.^6^,^8^,^9^ Since the late 1990s, at least 6 months of IPT has been recommended by the WHO for HIV-positive individuals. Guidelines from 2015 now recommend no less than 36 months for people without symptoms of active TB, irrespective of CD4 count, availability of TST, ART status or pregnancy.^10–12^ In high TB incidence, resource-constrained settings, absence of current cough, fever, night sweats and weight loss can be used as a screening tool for IPT eligibility.^13–16^ IPT provision should be supported by regular adherence support and monitoring, and ideally accompanied by pyridoxine to reduce the risk of symptomatic peripheral neuropathy.^17^

Barriers to the uptake and completion of IPT have been widely reported, and include longer regimens,^18^,^19^ lack of health worker training and patient education on treatment guidelines,^18^,^20^ distance from the clinic and incompletely integrated HIV-TB care.^20^,^21^ In Malawi, the first wide-scale use of IPT by the national HIV programme was not implemented until 2015.^15^,^22^ Lack of nationwide implementation was initially driven by concerns from the National TB Programme around the theoretical risk of inducing INH resistance, given the difficulties of excluding active TB disease, and historically low completion rates under stand-alone IPT services.^21^,^23^ Experience, however, suggests little impact on drug resistance patterns in practice.^15^ From the perspective of HIV programmes, ongoing concerns have included the cost and logistics of adding one (INH) or two (plus pyridoxine) drugs to already overstretched HIV care programmes, with the increasing pill burden and side-effect profile potentially having a deleterious impact on ART adherence, and the limited evidence of broader health benefits beyond TB prevention. Understanding the factors associated with non-completion of IPT could therefore help accelerate wider implementation, and inform strategies to improve both the uptake and completion of IPT at the programme level.

The main aims of the present study were to estimate non-completion of IPT and determine its predictors among HIV-positive adults initiating a 6-month course of IPT delivered in urban Blantyre by a research project before national roll-out.

## MATERIALS AND METHODS

### Study design and participants

This was a prospective cohort study nested within the intervention arm of a community cluster randomised trial of TB prevention (HitTB: ISRCTN02004005) conducted between February 2012 and June 2014. In the parent trial, community-based HIV testing and TB active case finding were offered in the 14 urban intervention communities comprising 16 660 adult residents, while 14 control communities received TB active case finding only. The trial profile and HIV testing procedures have previously been described in detail.^24^,^25^

In brief, participants who tested HIV-positive in the community were referred to their nearest primary care clinic for confirmatory HIV testing and counselling (HTC), WHO clinical staging assessment, CD4 count measurement, and linkage to HIV and TB care and prevention services, including IPT, all of which were completed by study nurses. Eligible participants for this cohort study were HIV-positive adults aged ⩾16 years who were resident within intervention neighbourhood clusters in urban Blantyre, Malawi, and who initiated IPT as part of trial interventions. During clinic assessment, participants were assessed for IPT eligibility using a symptom screening tool, with the presence of any of cough, fever, weight loss or night sweats prompting clinical assessment and investigation for active TB. Participants without TB symptoms and weighing ⩾35 kg were offered 300 mg INH and 25 mg pyridoxine once a day for 6 months to minimise the risk of INH peripheral neuropathy. IPT was, however, stopped in patients who developed neuropathy without attempting to distinguish the causative agent.

### Follow-up and outcome measurement

IPT was dispensed separately from ART, such that patients also receiving ART had to visit a second clinic room within the same facility for their repeat IPT prescriptions and final follow-up on months 1, 2, 3, 4, 5 and 6. IPT follow-up visits were harmonised as far as possible with other HIV care clinic appointments, but in some instances a special IPT visit was required. Adherence to IPT was monitored through monthly prescription refill records. During IPT clinic visits, participants were screened for TB symptoms (and investigated if required), and assessed for adverse events. The main emphasis was on early detection of clinical hepatitis and peripheral neuropathy through systematic enquiry for jaundice, nausea or vomiting, or any symptoms of peripheral neuropathy. All toxicities that led to IPT being temporarily or permanently discontinued, and all grade 3 or 4 toxicity effects,^26^ were recorded in participant-held treatment cards as well as in clinic registers.

Participants with serious adverse events (SAEs) were referred to Queen Elizabeth Central Hospital, Blantyre, for clinical management. SAEs and other adverse events were documented and reported to the Malawi College of Medicine Research Ethics Committee (COMREC), Blantyre, by the principal investigator (PI). Participants who died were followed up by verbal autopsy through a community liaison system of the parent trial, to ascertain the cause of death. IPT was given on a monthly basis, and participants who did not make all six clinic visits to refill INH due to loss to follow-up (LTFU), death, active/presumptive TB or having stopped treatment for any other reason, were classified as not having completed IPT. Home tracing for participants lost to follow-up was not conducted.

### Data collection and analysis

Study participant IPT registers were used to record baseline demographics, clinical characteristics and monthly outcomes. Data from the IPT registers were extracted using an optical character-recognition system, and imported into the study database. Descriptive statistics were used to characterise study participants, and to estimate the proportion of participants lost from IPT, stratified by clinical and socio-demographic characteristics. Age, sex, baseline WHO stage, CD4 count, ART status, provider of HTC service, reported past anti-tuberculosis treatment, year of IPT initiation and side effects were considered to be potential predictors of non-completion of IPT. All potential predictors were evaluated for inclusion in a multivariate model,^27^ with age and sex included a priori. The final multilevel logistic regression model adjusted for patient characteristics, and a random-effect term to account for clustering by participant neighbourhood of residence.^25^ Analysis was carried out using Stata v14.0 (StataCorp, College Station, TX, USA) and *R* v3.3.1 (R Foundation for Statistical Computing, Vienna, Austria).

### Ethics consideration

The parent study protocol was approved by the COMREC, Blantyre, and the London School of Hygiene & Tropical Medicine, London, UK. Participants provided written (or witnessed thumbprint) informed consent for HIV testing interventions, and verbal consent to initiate IPT as part of routine clinical care.

## RESULTS

### Characteristics of study participants

Of the 16 660 adult residents of the 14 intervention clusters, 14 004 (84.1%) underwent study HIV testing and 1725 (12.3%) were confirmed to be HIV-positive. Of the 1557/1725 (90.3%) HIV-positive participants screened for IPT eligibility, 1301 (83.6%) met the IPT eligibility criteria. Non-screening of 168 individuals was due to unavailability. Non-eligibility of those screened was due to presumptive TB (212/256, 82.8%), active TB (14/256, 5.5%), epilepsy (11/256, 4.3%), high alcohol intake (10/256, 3.9%), previous reaction to IPT (6/256, 2.3%) and known liver disease (3/256, 1.2%). A further 17 participants declined IPT, resulting in an IPT initiation rate of 1284/1301 (98.7%) among eligible participants (Figure).

**Figure:**
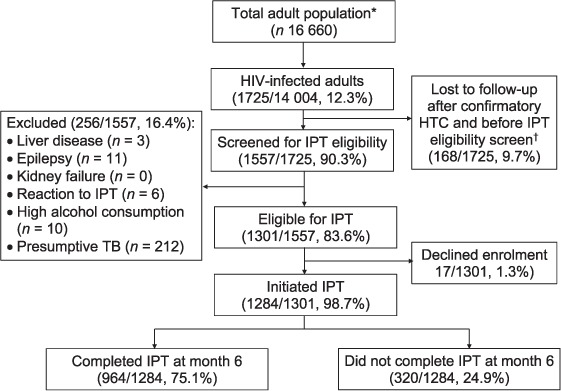
Flow of study participants enrolled for IPT. ^*^ Not adjusted for migration. ^†^ Confirmatory HTC using finger-prick parallel rapid diagnostic testing by study nurse following patient home-based HIV self-testing. HIV = human immunodeficiency virus; IPT = isoniazid preventive therapy; HTC = HIV testing and counselling; TB = tuberculosis.

The mean age of the participants was 35.1 years (standard deviation ±10.2, interquartile range [IQR] 28–40; *n* = 1276); the median CD4 count was 337 cells/μl (IQR 199–511; *n* = 1114). Most participants were female (885/1280, 69.1%), 25 (2.8%) of whom were pregnant. One hundred and eleven participants (8.9%) had previously been treated for TB: 8/111 (7.2%) in the past 2 years and 103/111 (92.8%) over 2 years previously (Table 1).

**Table 1:**
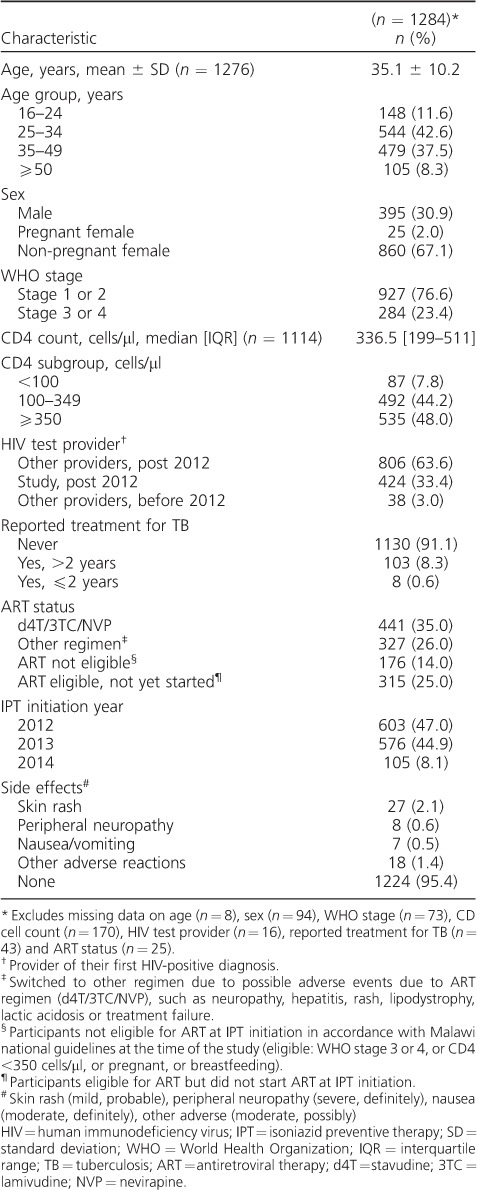
Demographic and clinical characteristics of HIV-positive participants who started IPT

### Initiation and completion of IPT

Of the 1284 IPT initiators, 320 (24.9%, 95% confidence interval [CI] 22.6–27.4) did not complete 6 months of therapy. Non-completion of IPT was due to LTFU (243/320, 75.9%), death (10/320, 3.1%), development of active/presumptive TB (4/320, 1.3%) and approved decision to discontinue IPT (63/320, 19.7%) (Table 2). Of the 63 participants whose IPT was discontinued, 50 (79.4%) were due to side effects, including peripheral neuropathy (12.0%), nausea/vomiting (12.0%), skin rash (46.0%) and other possible adverse reactions (30.0%). The reason for discontinuing IPT was not recorded for 13 participants.

**Table 2:**
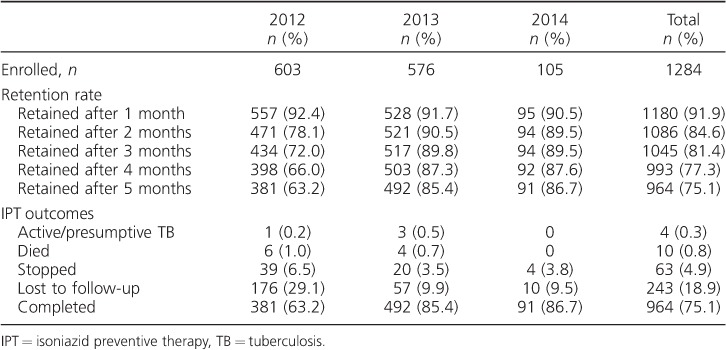
Retention rates (cumulative percentage) and outcomes of patients enrolled on IPT in Blantyre, Malawi

Approximately half of this large cohort study comprised HIV-positive participants who were started on cotrimoxazole as well as a first-line ART regimen (stavudine/lamivudine/nevirapine [d4T/3TC/NVP]), which is known to have side effects that overlap with those of INH, notably peripheral neuropathy, hepatitis and nausea. IPT was generally safe and well tolerated, with only 60 (4.6%) participants reporting side effects. Eight patients developed peripheral neuropathy, all of whom had also recently been initiated on d4T-containing ART regimens. Other side effects, including skin rash, were frequent and non-severe. Grade 2 nausea and vomiting were the least observed IPT side effects. There was no case of severe hepatitis. Verbal autopsy of 10 participants who died during treatment did not suggest any relationship to IPT.

Comparing the years of IPT implementation, a sharp decrease in the proportion of participants failing to complete IPT occurred in those initiating treatment from 2012 (222/603, 36.8%) to 2013 (84/576, 14.6%) and 2014 (14/105, 13.3%). Overall, LTFU was the most common reason for non-completion of IPT in all years (Table 2).

### Risk factors for non-completion of IPT

In univariate analysis, WHO stage 3/4, CD4 count <100 cells/μl, CD4 count 100–349 cells/μl, history of anti-tuberculosis treatment and having side effects were independently associated with non-completion of IPT; participants with WHO stage 3/4 had just over 3-fold increased odds of not completing IPT compared with those in WHO stage 1/2 (odds ratio [OR] 3.13, 95%CI 2.35–4.16). Participants with CD4 count <100 cells/μl or 100–349 cells/μl were more likely not to complete IPT than those with CD4 count ⩾350 cells/μl (OR 2.21, 95%CI 1.36–3.58 and OR 1.48, 95%CI 1.11–1.98, respectively). Furthermore, participants who had been treated for TB over 2 years previously were more likely not to complete IPT than those without a history of anti-tuberculosis treatment (OR 1.56, 95%CI 1.01–2.42). There was a 23-fold greater risk of non-completion of IPT among participants who experienced side effects than among those without side effects (OR 23.3, 95%CI 10.90–49.69) (Table 3).

**Table 3:**
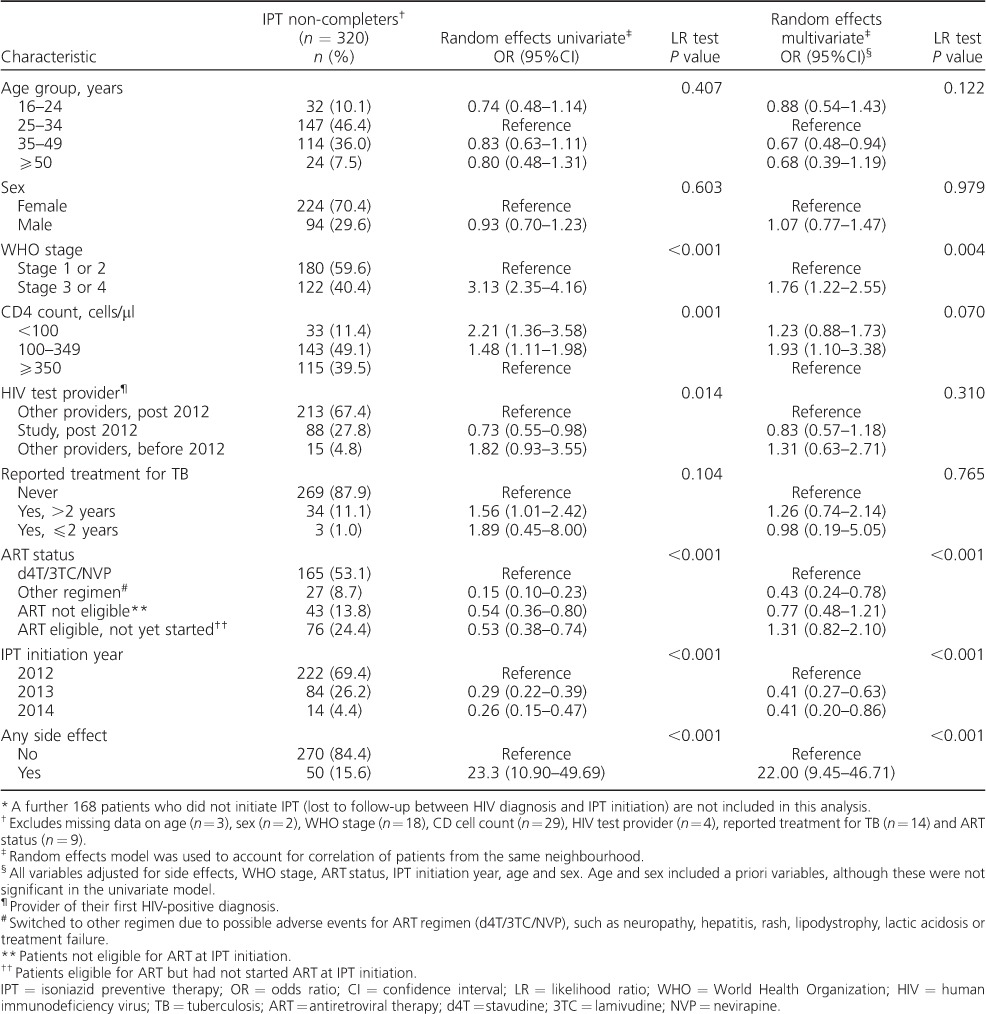
Non-completion of IPT at month 6 (using univariate and multivariate random effect models) ^*^

In multivariate analysis, the following variables remained significantly associated with non-completion of IPT: WHO stage 3/4 (adjusted OR [aOR] 1.76, 95%CI 1.22–2.55, *P*=0.004), CD4 count 100–349 cells/μl (aOR 1.93, 95%CI 1.10–3.38, *P*=0.024) and reporting side effects (aOR 22.00, 95%CI 9.45–46.71, *P* < 0.001) (Table 3).

## DISCUSSION

A quarter of the adults diagnosed as HIV-positive did not complete IPT in this prospective cohort study, which recruited participants from urban communities of Blantyre. Independent risk factors for non-completion of IPT included being in WHO stage 3 or 4, having a CD4 count of <350 cells/μl and having side effects due to INH. Most non-completion (75.9% of 320 non-completers) was due to LTFU for unknown reasons.

The highest risk period for LTFU was immediately after confirmatory HIV testing, but before assessment for IPT eligibility (168/1725, 9.7%). The period immediately following diagnosis is known to be a high-risk period for loss to health services for other conditions, such as TB, as well as for HIV testing in routine clinics. National HIV programmes should focus on supporting newly diagnosed patients to remain in care, and they should streamline and integrate IPT eligibility assessments as far as possible. The completion rate of 75.1% reported in the present study is much higher than estimates from several other studies,^18^,^28^,^29^ but lower than in two studies nested within the DarDar trial in Tanzania^21^,^23^ and a study in Zimbabwe.^4^ This finding suggests that setting-specific factors, including the configuration of joint HIV-TB services, have an important influence on completion of IPT, underscoring the importance of studies such as this one. While those aged <30 years and females have been shown to be less likely to complete IPT in previous studies in Africa,^23^,^29^ we did not find these characteristics to be associated with non-completion of IPT in the present study, which provided IPT separately from other routine HIV care, but at the primary care level.

Our data show evidence of programmatic learning and the importance of an accompanying ART regimen, with substantially lower discontinuation rates in 2014 than in 2012. This is likely due to the combination of growing prescriber confidence from familiarity with IPT and a programme switch to a better tolerated first-line ART regimen (tenofovir/lamivudine/efavirenz, TDF/3TC/EFV) from February 2014. It should be noted that the previous d4T-containing first-line ART regimen (d4T/3TC/NVP) was the most likely cause of peripheral neuropathy in patients started on both ART and IPT (as pyridoxine was provided to minimise INH-related peripheral neuropathy). However, to simplify subsequent ART management, we elected to manage these events pragmatically by discontinuing IPT and referring patients for alternative first-line ART.

Limitations of the present study included use of self-reported adherence and pill counts, which are less sensitive measures of adherence than, for example, urine drug tests or electronic Medication Event Monitoring System devices. Our completion rates could therefore have been overestimated. Other characteristics, such as marital status, education levels, employment status, religion and ethnicity, play roles in treatment adherence, as reported by other studies,^28^,^29^ and were not captured in the present study.

The main strength of our study was that potential predictors of non-completion of IPT were determined before ascertaining the outcome, thereby minimising information bias. Unrecognised deaths have comprised a substantial fraction of LTFU in other studies, and could explain the higher rates of non-completion among immunosuppressed individuals reported here. However, we think this is unlikely, as the parent study had systematic reporting of all deaths in the community with follow-up verbal autopsy. An alternative explanation could be that participants with lower CD4 counts may have been more likely to migrate out of this urban slum setting if unable to work due to ill health.

The main policy implications of our findings are to underscore the increased risk of loss to TB prevention services in the period immediately following HIV diagnosis. Our data also highlight the importance of early diagnosis of HIV and prompt ART and IPT initiation,^30^ as the more severely immunosuppressed HIV-positive adults were at greater risk of failing to complete IPT. A number of interventions, including automated mobile phone short message services (SMS), smart-phone applications and home visits by lay counsellors, could be considered to provide extra support to help retain high-risk individuals in their first months of HIV care.^31^ IPT guidelines would benefit from detailing the health messages and information needed by individuals initiating IPT to specifically address the potential side effects for patients starting both ART and IPT.

In summary, completion of 6 months of IPT among HIV-positive adults from poor urban communities in Malawi was suboptimal, but better than observed in several other primary care clinic settings. IPTwas well tolerated, especially in the year after the national switch away from a d4T-containing first-line ART regimen. Interventions to further improve retention should target the period immediately after HIV diagnosis, with support for the more immunosuppressed patients, as well as providing treatment literacy on the benefits of IPT and known side effects. Programmes offering IPT through clinics that are not fully integrated into other pre-ART and ART services should also focus on supporting patients to complete their first and second months of IPT initiation, as most patients drop out at these stages.
